# Production of 1,3-PDO and butanol by a mutant strain of *Clostridium pasteurianum* with increased tolerance towards crude glycerol

**DOI:** 10.1186/2191-0855-2-44

**Published:** 2012-08-17

**Authors:** Torbjørn Ølshøj Jensen, Thomas Kvist, Marie Just Mikkelsen, Peter Westermann

**Affiliations:** 1 Section for Sustainable Biotechnology, Aalborg University, Copenhagen, A.C. Meyers Vaenge 15, DK-2450, Copenhagen SV, Denmark; 2 Biogasol ApS, Lautrupvang 2A, DK-2750, Ballerup, Denmark

**Keywords:** Glycerol, Crude glycerol, Biofuel, Anaerobic fermentation, Chemical mutagenesis

## Abstract

The production of biodiesel results in a concomitant production of crude glycerol (10% w/w). *Clostridium pasteurianum* can utilize glycerol as sole carbon source and converts it into 1,3-propanediol, ethanol, butanol, and CO_2_. Reduced growth and productivities on crude glycerol as compared to technical grade glycerol have previously been observed. In this study, we applied random mutagenesis mediated by ethane methyl sulfonate (EMS) to develop a mutant strain of *C. pasteurianum* tolerating high concentrations of crude glycerol. At an initial crude glycerol concentration of 25 g/l the amount of dry cell mass produced by the mutant strain was six times higher than the amount produced by the wild type. Growth of the mutant strain was even detected at an initial crude glycerol concentration of 105 g/l. A pH controlled reactor with *in situ* removal of butanol by gas-stripping was used to evaluate the performance of the mutant strain. Utilizing stored crude glycerol, the mutant strain showed significantly increased rates compared to the wild type. A maximum glycerol utilization rate of 7.59 g/l/h was observed along with productivities of 1.80 g/l/h and 1.21 g/l/h of butanol and 1,3-PDO, respectively. These rates are higher than what previously has been published for *C. pasteurianum* growing on technical grade glycerol in fed batch reactors. In addition, high yields of the main products (butanol and 1,3-PDO) were detected and these two products were efficiently separated in two steams using gas-stripping.

## Introduction

Limited availability of fossil resources and increasing global impact from the release of fossil fuel-derived CO_2_ has increased the development of biological production of renewable alternatives. Biodiesel production from plant lipids is considered a renewable alternative to mineral oil-derived diesel ([Demirbas 2007]). The increasing market for biodiesel has substantially altered the cost and availability of glycerol released from transesterification of fatty acids from lipids. Without purification, this glycerol (crude glycerol), is considered a waste ([Johnson and Taconi 2007]; Dobson et al. [2012]) and it is, therefore, important that new and sustainable solutions for utilization of the crude glycerol are developed (Pyle et al. [2008]; [Yazdani and Gonzalez 2007]; [Willke and Vorlop 2008]).

The major microbial conversion route of glycerol leads to the production of 1,3-propanediol (1,3-PDO) (Pagliaro et al. [2007]). Currently, 1,3-PDO is used as a diol component in the plastic poly-trimethylene terephthalate (PTT), which is comparable to nylon ([Willke and Vorlop 2008]). PTT is mainly used for carpet and textile fiber production (DuPont™ Sorona® [2011]). The organisms best known for producing 1,3-PDO are *Clostridium butyricum* and *Klebsiella pneumoniae*, which both can achieve high yields and productivities (Biebl et al. [1999]; Zeng et al. [1994]).

Since the 1,3-PDO pathway does not lead to ATP production, other pathways are needed for the generation of energy. The formation of acetate and/or butyrate leads to ATP production but is also associated with the generation of reducing equivalents, which are regenerated by the 1,3-PDO pathway (Rossi et al. [2012]; [Zeng 1996]).

The most commonly used strategy for 1,3-PDO production is fed-batch fermentation, because it combines high product concentrations with a low excess of glycerol in the fermentation broth, which both are critical for the downstream processing ([Zeng and Biebl 2002]). However, accumulation of by-products such as acetate, butyrate, or ethanol can cause inhibition of the organism ([Biebl 1991]).

Butanol is an important bulk chemical for the synthesis of a variety of chemical products and an efficient biofuel with properties clearly superior to ethanol such as lower enthalpy of vaporization, lower solubility of water, less corrosiveness, and a much higher energy density (Dürre [2008]).

The production of butanol and 1,3-PDO from biodiesel derived glycerol will not only constitute a sustainable utilization of waste glycerol for fuel production, but also a means to produce two chemicals, which can be used in the chemical industry.

The most studied organism for biological production of butanol is *C. acetobutylicum*. However*, C. acetobutylicum* cannot grow solely on glycerol as it cannot re-oxidize the excess NADH generated in the cellular glycerol catabolism. (Girbal et al. [1995]; Gonzalez-Pajuelo et al. [2005])

In contrast to *C. butyricum* which produces 1,3-PDO along with the formation of butyrate and acetate, *C. pasteurianum* produces butanol, 1,3-PDO, and ethanol as main products. Fermentation of glycerol by *C. pasteurianum* was firstly described by Nakas et al. ([1983]) in an attempt to obtain a marketable product from photosynthetically produced glycerol from halophilic algae.

Only few publications have dealt with optimization of glycerol fermentation by *C. pasteurianum* ([Biebl 2001]; Dabrock et al. [1992]). Recently, a study demonstrating the possibility of utilizing crude glycerol in fermentative butanol production has been published (Taconi et al. [2009]). However, growth and productivity were significantly lower compared to results obtained from fermentation of pure glycerol. In order to achieve a high productivity, upstream purification has been successfully applied (Venkataramanan et al. [2012]; Jensen et al. [2012]). Another approach is to develop strains capable of coping with the toxicity of the crude glycerol. To construct/develop such an organism a directed or a random approach can be used. Even though directed strain improvement by systems metabolic engineering may be necessary as reported by (Lee et al. [2005]) and Lee et al. ([2008]) the lack of a complete genome sequence and adequate tools limits the application on *C. pasteurianum*.

In this study we have, therefore, chosen to develop a strain of *C. pasteurianum* with elevated tolerance towards to crude glycerol derived from the biodiesel production by use of chemical mutagenesis and selection.

## Materials and methods

### Bacterial strain

The strain *C. pasteurianum* (DMSZ 525) was purchased from the German Collection of Microorganism and Cell Cultures (DSMZ), Göttingen, Germany.

### Medium and conditions

The minimal medium used for both reactor and 10 ml batch fermentations was described by Jensen et al. ([2012]). When solid media were used, 15 g/l agar was added to the media prior to autoclavation. All cultures were incubated anaerobically at 37°C at pH 6.0 under a gas phase of N_2_/CO_2_ (80:20). The carbon source was either purified (technical grade) glycerol (Sigma-Aldrich, St. Louis, Missouri, USA) or biodiesel derived crude glycerol (Meroco, Leopoldov, Slovakia). The crude glycerol was derived from 100% rape seed oil and was specified to contain 800–850 g/l glycerol, less than 1 g/l methanol, 55 g/l NaCl and 2.5**%** MONG (matter organic non glycerol) by the manufacturer. All amounts of crude glycerol presented are based on measured concentrations.

### Chemical mutagenesis and evolutionary adaption

A fresh *C. pasteurianum* culture (50 or 100μL) was plated on petri dishes containing solid minimal medium supplemented with 50 g/l and 70 g/l crude glycerol. A drop (approximately 7 μl) of ethane methyl sulfonate (EMS) was placed in the middle of the plates. The plates were incubated at 37°C for three days in an anaerobic jar together with a humid anaerocult® A (Merck, Darmstadt, Germany). An inoculated plate (without EMS) was incubated as a control in the jar.

After incubation, single colonies were picked from the edge of the clearing zones caused by EMS. The selected colonies were streaked on plates with similar or higher glycerol concentration without EMS. When growth was detected, the colonies were repeatedly streaked on plates with similar or higher glycerol concentration to increase the tolerance towards the crude glycerol. The crude glycerol concentration in the medium was; 50, 60, 70, 80, 90, 100, and 110 g/l.

Colonies able to grow on 110 g/l crude glycerol plates were inoculated in liquid minimal medium supplemented with 25 g/l crude glycerol. The liquid culture was used as inoculums for the toxicity test.

### Treatment of the crude glycerol

Glycerol was stored at room temperature for 10 months as previously described (Jensen et al. [2012]. Approximately 0.05 g of activated stone carbon 0.4 - 0.85 mm per 10 ml of medium (Gert Strand, Malmö, Sweden) was added prior to autoclavation (Jensen et al. [2012]).

### Toxicity test

To assess the inhibition of the glycerol, toxicity tests were performed in batch experiments. A series of vials were prepared with different concentrations of glycerol ranging from 10 to 200 g/l. The vials were inoculated with 0.2 ml of an overnight culture. After 20 hours of incubation, growth was evaluated based upon the amount of dry cell mass. The dry cell mass was determined spectrophotometrically after establishing a linear correlation between the dry cell mass and cell suspension absorbance at 595 nm. All tests were conducted in at least duplicates.

### Reactor fermentation

The reactor fermentation experiments were carried out as described by Jensen et al. ([2012]) in 500 ml glass reactors at 37°C. The active volume was 400 ml and the inoculation volume was 10%. pH was maintained at 6.0 by addition of 1 M KOH. Mixing was performed by magnetic stirring. For *in situ* removal of solvent, gas-stripping was applied, circulating the produced gas by means of a peristaltic pump at a flow rate of approximately 600 ml/min.

For stoichiometric calculations the composition of cell biomass was assumed to be CH_1.65_ N_0.23_O_0.45_ (Hild et al. [2003]).

### HPLC analysis

Liquid samples were analyzed for glycerol, lactate, acetate, 1,3-PDO, butyrate, ethanol, acetone, and butanol using high-pressure liquid chromatography. The HPLC system was equipped with a Rezex ROA-Organic Acid column (Phenomenex, Torrance, California, USA) and a RI 101 refractive index detector (Shodex, Kawasaki, Japan). The mobile phase was 4.5 mM H_2_SO_4_, pumped at a flow-rate of 0.6 mL/min. Prior to the analysis the samples were centrifuged at 12,000 x g for 10 minutes. The supernatant was separated and diluted to a suitable concentration range before it was loaded on the HPLC.

## Results

### Strain development

**Figure 1:**
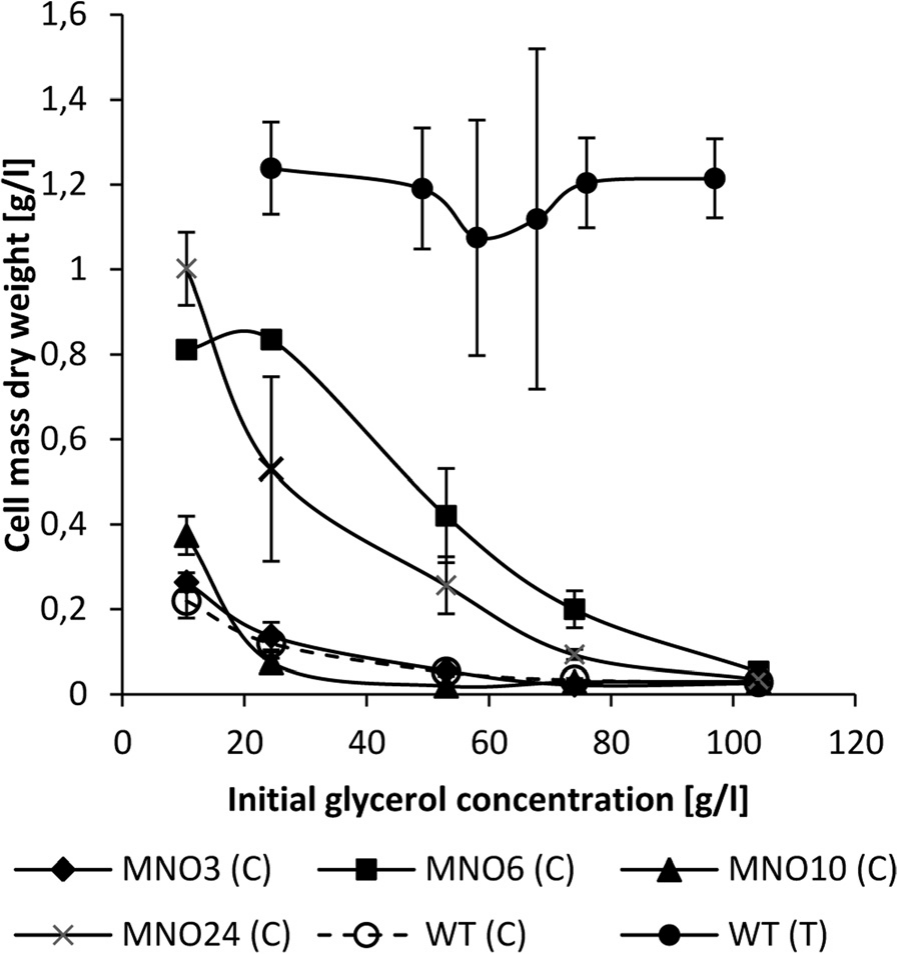
**Toxicity test of 4 selected mutant strains and the wild type (WT).** Incubation for 20 hours, the error bars indicate the standard deviation. (C): Crude glycerol; (T): Technical grade glycerol. Data of the WT grown on technical grade glycerol derived from Jensen et al. ([2012]).

After incubation in the presence of EMS for 3 days, colonies appeared only on the plates with the lowest initial crude glycerol concentration (50 g/l). A clearing zone was present on the plates around the droplet of EMS. In total, 37 colonies from the edge of the clearing zone were picked and streaked on plates with 50 g/l glycerol serving as a strain library. Repeated transfers of the colonies to plates with increasingly higher glycerol concentrations enhanced the tolerance to the crude glycerol further. However, only few of the 37 colonies were able to grow at a crude glycerol concentration of 110 g/l. The most promising four strains were benchmarked in a toxicity test towards the wild type strain (Figure 1). At an initial glycerol concentration of 25 g/l the amount of dry cell mass of MNO24 was 3.5 times higher compared to the wild type on crude glycerol, however, with a considerable deviation. At the same initial glycerol concentration, MNO6 grew better than the wild type, producing 6 times the amount of dry cell mass. Growth characteristics by the mutant strains MNO3 and MNO10 were almost similar to the wild type. At an initial crude glycerol concentration of 53 g/l growth by the wild type, MNO3, and MNO10 was almost negligible, while MNO6 and MNO24 were less inhibited than the wild type grown on technical grade glycerol. At an initial glycerol concentration of 75 g/l, MNO6 produced 16.5% dry cell mass compared to the wild type growing on technical grade glycerol. The amount of dry cell mass produced by MNO24 constituted 7.7% of the amount produced by the wild type on technical grade glycerol while the wild type on crude glycerol only yielded 2%. At an initial glycerol concentration of 105 g/l, growth of MNO6 was detectable. Based on these results strain MNO6 appeared most tolerant to crude glycerol and was, therefore, chosen for the subsequent experiments.

**Figure 2:**
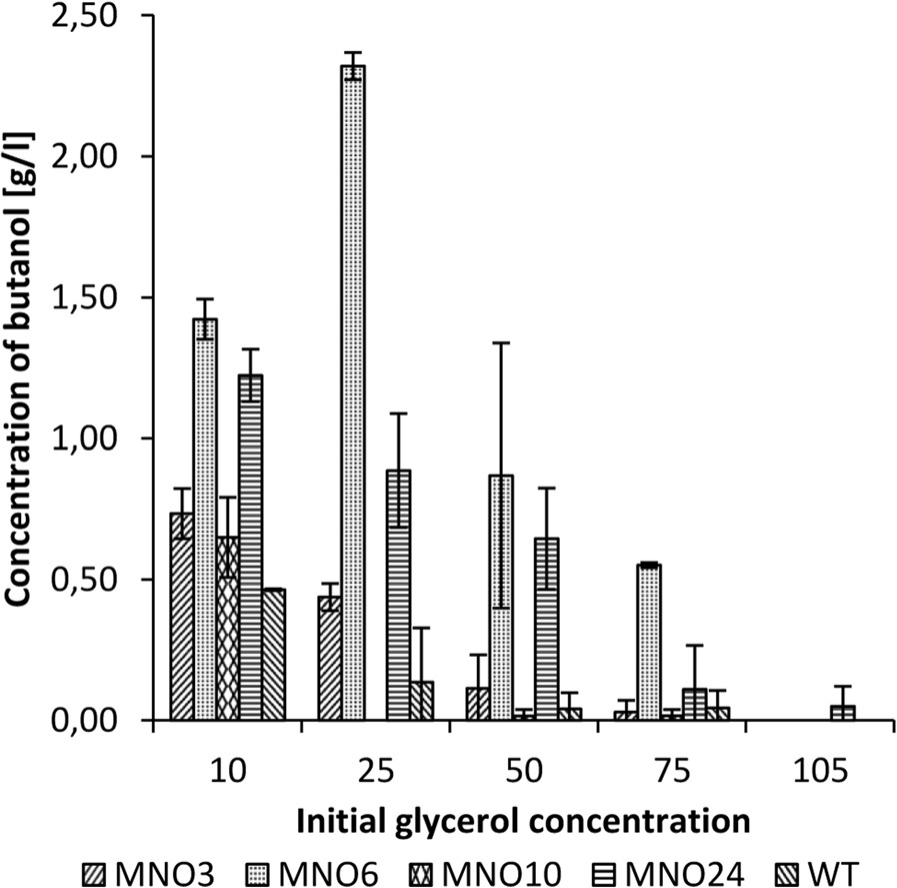
**Histogram showing the concentration of butanol at different initial crude glycerol concentrations after 20 hours of incubation.** The error bars indicate the standard deviation.

Chemical mutagenesis selecting for increased tolerance may lead to down-regulation of the genes responsible for the desired products ([Borden and Papoutsakis 2007]). Therefore, the product concentration at different initial glycerol concentrations was measured (Figure 2). Strain MNO6 exhibited the highest growth and achieved the highest concentration of butanol. At 25 g/l, strain MNO6 produced 2.32 g/l butanol while the wild type only produced 0.14 g/l. This demonstrates that the enhanced tolerance by MNO6 was neither achieved at the expense of butanol formation nor by product degradation. 

**Figure 3:**
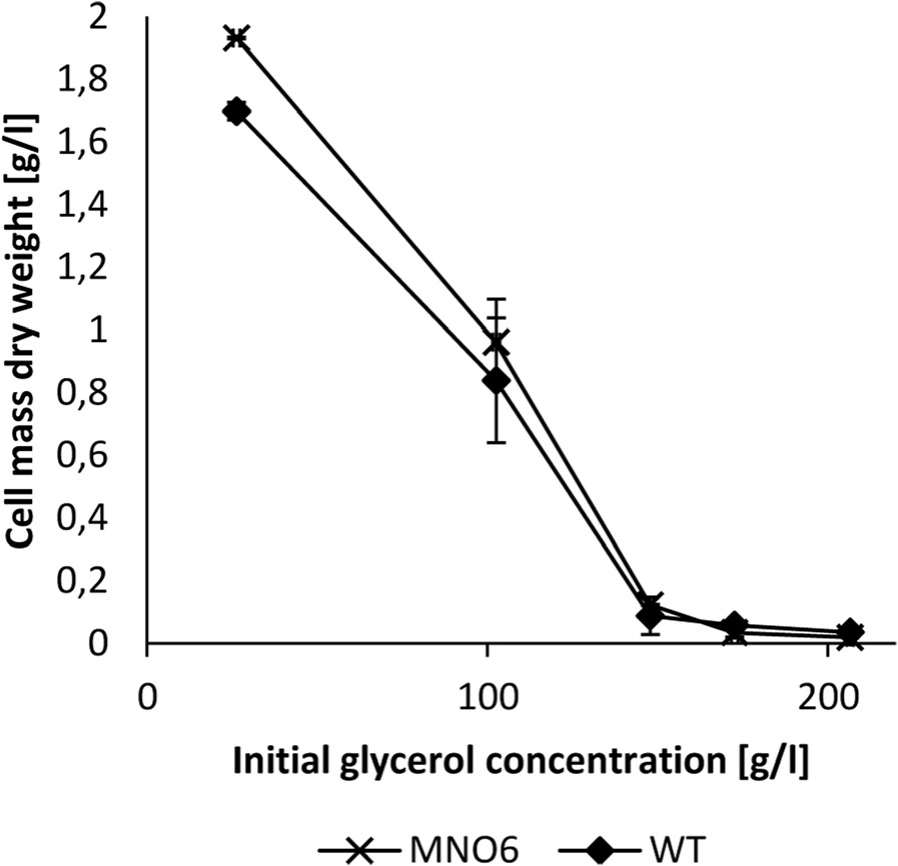
**Toxicity test of strain MNO6 and the wild type grown on stored crude glycerol supplemented with activated stone carbon.** Incubation for 20 hours, the error bars indicate the standard deviation.

We have previously found that growth on crude glycerol was highly supported by storage of the glycerol combined with supplementation of activated stone carbon (Jensen et al. [2012]). To assess growth at these conditions, strain MNO6 was benchmarked against the wild type in a toxicity test (Figure 3). As expected, the amount of dry cell mass produced by both strains on treated crude glycerol was higher compared to the amount produced on non-treated crude glycerol, and even higher than the amount produced by the wild type on technical grade glycerol (Figure 1). Compared to the wild type, MNO6 was less inhibited by the treated crude glycerol, as more cell mass was produced. However, the difference between the strains was only significant at 25 g/l (p = 0.047). 

### Fermentation

**Figure 4:**
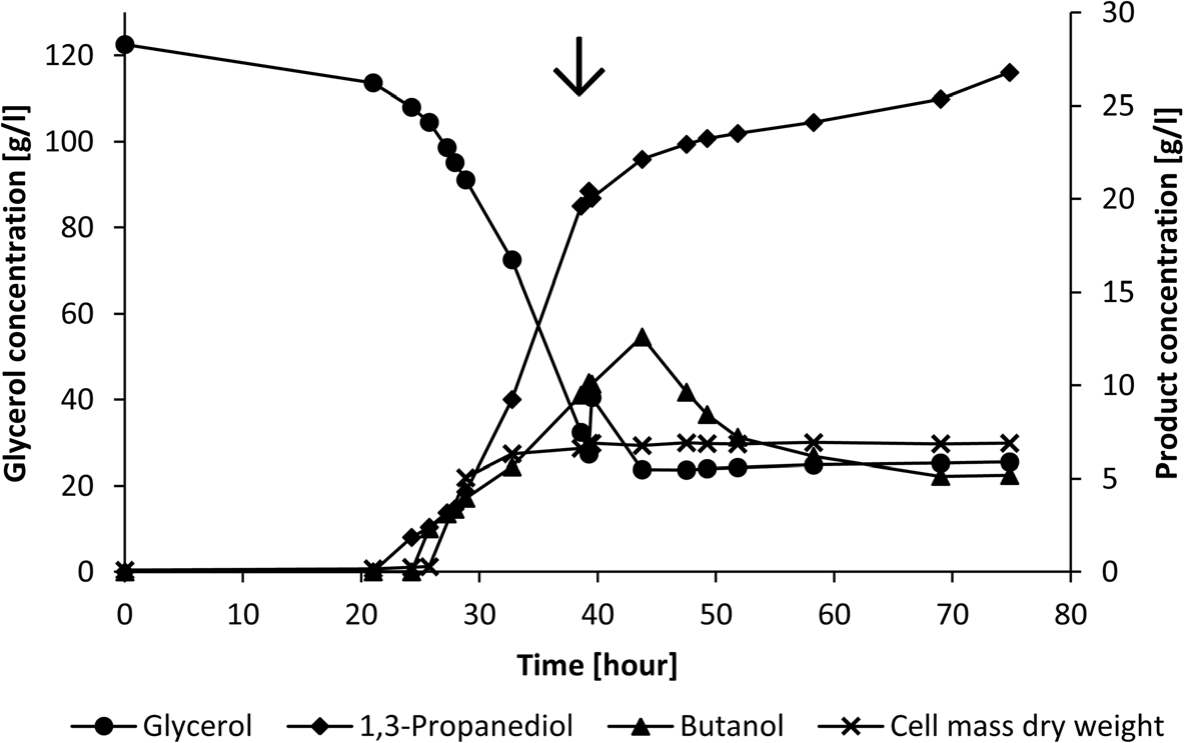
**Profile of the fermentation of stored crude glycerol supplemented with activated stone carbon by MNO6.** The initial glycerol concentration was 122 g/l, pH was maintained at 6, and the temperature was 37°C. Butanol concentration was measured in the fermentation broth. The arrow indicates the addition of 25 ml glycerol (250 g/l).

To assess the fermentation capabilities of MNO6 under pH controlled conditions, a reactor fermentation was set up utilizing stored crude glycerol supplemented with activated stone carbon. MNO6 was not tested on technical grade glycerol as it was irrelevant for industrial applications. The fermentation profile is shown in Figure 4. During the initial 21 hours, only a slow glycerol consumption was observed. After 21 hours the consumption of glycerol increased until a maximum rate of 7.59 g/l/h was reached after 39 hours of incubation. In order to maintain a sufficient amount of substrate, 25 ml glycerol (250 g/l) was added shortly after the maximum rate had been achieved. After this, too high amounts of butanol were produced and the gas stripper could no longer maintain the concentration below the toxic level. After 43.75 hours butanol concentration reached 12.6 g/l which arrested the metabolism of MNO6. When no production occurred, the removal rate by the gas-stripper was estimated to 0.79 g/l/h at the observed butanol concentration. Accounting for butanol removal, the maximum butanol productivity was calculated to 1.80 g/l/h. In the same period 1,3-PDO productivity was 1.21 g/l/h. Although, no butanol was produced for a considerable period, it was not possible to reduce butanol concentration below 5 g/l with this reactor-setup. After 75 hours, gas circulation was stopped and the products were quantified. Since, the activity was unaffected by addition of glycerol at t = 39.25 h and the activity did not resume when the butanol concentration was reduced below 6 g/l, at which the wild type regain metabolic activity (Jensen et al. [2012]), a lack of nutrients have probably caused the discontinuation. 

A mass-balance in mol based on the measured products was established:

(1) $$C_{3}H_{8}O_{3}\to 0.251\;C_{3}H_{8}O_{2}+0.036\;C_{2}H_{4}O_{2}+0.018\;C_{2}H_{6}O+0.020\;C_{4}H_{8}O_{2}+0.252\;C_{4}H_{10}O+0.559\;CO_{2}+0.388\;H_{2}+0.466\;H_{2}O+0.184\;\mathrm{cell}-\mathrm{biomass}$$

The strain did not produce any lactate, and only very low amounts of acetate, ethanol, and butyrate. The carbon recovery was only 87%. The missing carbon could be butanol, as accumulation of butanol in the gas collection bag was observed. Adding the gas-phase products to the mass balance leads to a butanol yield of 0.296 mol/mol and a CO_2_ yield of 0.687 mol/mol. The butanol yield achieved represents 59.2% of the theoretical maximum based on the stoichiometry:

(2) $${2\text{C}}_{3}{\text{H}}_{8}{\text{O}}_{3}\to {\text{C}}_{4}{\text{H}}_{10}\text{O}+{2\text{CO}}_{2}+2{\text{H}}_{2}+{\text{H}}_{2}\text{O}$$

The two main compounds 1,3-PDO and butanol were produced in almost equimolar amounts with a 18% surplus of butanol after the mass-balance had been adjusted.

## Discussion

Random mutagenesis is a generally an accepted and widely used approach for increasing tolerance of a bacterial strain towards an unknown inhibitor. EMS and N-methyl-N-nitro-N-nitrosoguanidine (MNNG), which both act by direct mutagenesis inducing base substitutions or deletions, have been used with success in the closely related *C. acetobutylicum* ([Annous and Blaschek 1991]; Elkanouni et al. [1989]). Recently, Malaviya et al. ([2012]) succeeded in developing an effective butanol producing mutant strain of *C. pasteurianum* by the use of MNNG. However, both [Lemmel (1985]) and Syed et al. ([2008]) obtained *C. acetobutylicum* mutants with higher tolerance and productivity using EMS and reported that MNNG was a less effective mutagenic compound. Our success with increased tolerance towards crude glycerol and increased butanol productivity by EMS mutagenesis confirmed the efficiency of EMS for development of *C. pasteurianum* mutants. As the mutant strain was developed by chemical mutagenesis it will not be classified as a Gene Modified Organism (GMO) reducing the operating costs and safety precautions necessary to run a GMO-based production.

We have previously examined yields and fermentation of stored crude glycerol supplemented with activated stone carbon by *C. pasteurianum* wild type (Jensen et al. [2012]). The fermentations by MNO6, performed in this study was done with the same crude glycerol as substrate and under almost similar conditions, and benchmarking of MNO6 against data from the wild type strain study, is therefore relevant (Table 1). 

**Table 1:** Comparison of fermentation data obtained in this study with a related study

	**Technical grade glycerol(Jensen et al.**[2012]**)**	**Stored crude glycerol + activated stone carbon (Jensen et al.**[2012]**)**	**Stored crude glycerol + activated stone carbon**
	***Wild type***	***Wild type***	***MNO6***
**Maximum Glycerol rate**	4.94 g/l/h	4.08 g/l/h	7.59 g/l/h
**Maximum 1,3-PDO rate**	0.64 g/l/h	0.91 g/l/h	1.21 g/l/h
**Maximum Butanol rate**	1.21 g/l/h*	1.30 g/l/h	1.80 g/l/h
**Butanol yield**	264 mmol/mol	280 mmol/mol	252 mmol/mol
**1,3-PDO yield**	217 mmol/mol	169 mmol/mol	251 mmol/mol
**Ethanol yield**	164 mmol/mol	55 mmol/mol	18 mmol/mol
**Carbon Recovery**	97%	88%	87%

*The rate was based on a fermentation without gas-stripping.

The maximum glycerol utilization rate attained by MNO6 was 7.59 g/l/h whereas the wild type strain reached rates of 4.08 g/l/h and 4.94 g/l/h utilizing stored crude glycerol and technical grade glycerol, respectively. This corresponds to an increased rate of 86% and 55% compared to the wild type on crude and technical grade glycerol. The production rates were similarly increased by 33% for 1,3-PDO and 46% for butanol compared to the wild type grown on technical grade glycerol. When the rates achieved with MNO6 were compared to rates of the wild type grown on similar glycerol, maximum 1,3-PDO productivity was increased by 89% and maximum butanol productivity was increased by 49%. Malaviya et al. ([2012]) demonstrated significantly increased production rates in a high cell density continuous bioreactor using a so-called hyper producing *C. pasteurianum* mutant strain. Under batch conditions the mutant strain had a 14% higher butanol productivity compared to the wild type. This is lower than the increase in productivity by MNO6 demonstrated in this study.

In a glucose fed batch bioreactor with gas-stripping, Ezeji et al. ([2004]) achieved a maximum butanol productivity of 1.81 g/l/h (65% of total solvent productivity) utilizing the well known ABE producing strain *C. beijerinckii* BA101. This is similar to the maximum butanol productivity achieved by MNO6. However, Ezeji et al. reached the productivity by utilizing pure glucose where MNO6 utilized stored crude glycerol.

In the mass balance the carbon recovery was 87% (Table 1). A similar carbon recovery was observed in the fermentation by the wild type utilizing stored crude glycerol supplemented with activated stone carbon (Table 1). We assume that the resulting carbon was non-condensed butanol in the gas-phase. Also, activated stone carbon has been considered as an adsorbent for butanol in downstream processing (Qureshi et al. [2005]; [Groot and Luyben 1986]). When fermentations were carried out without activated stone carbon (on technical grade glycerol) a higher carbon recovery was observed (Table 1).

After adjusting the mass balance, the yield of butanol from stored crude glycerol by MNO6 was 0.296 mol/mol compared to 0.264 mol/mol by the wild type utilizing technical grade glycerol. Venkataramanan et al. ([2012]) reported a butanol yield of 0.347 mol/mol by *C. pasteurianum* utilizing purified crude glycerol and 0.322 mol/mol when technical grade glycerol was used. Both yields are higher than those achieved by MNO6 and the wild type. Low initial glycerol concentration, low utilization of glycerol as well as other dissimilarities in growth conditions in the two studies, could cause this difference.

Strain MNO6 had a 1,3-PDO yield of 0.249 mol/mol, which is 47% higher than the yield by the wild type (0.169 mol/mol) grown on stored crude glycerol. Even though the 1,3-PDO yield was lower than the theoretical maximum of 0.720 mol/mol ([Zeng 1996]) MNO6 has an interesting potential, as 1,3-PDO is produced simultaneously with butanol. Productivity of 1,3-PDO in fed batch has been reported to 0.9-3.0 g/l/h by the dedicated 1,3-PDO producer *C. butyricum* ([Willke and Vorlop 2008]; Chatzifragkou et al. [2011]; Wilkens et al. [2012]; [Reimann and Biebl 1996]). The achieved maximum 1,3-PDO production rate of 1.21 g/l/h by MNO6 is comparable to *C. butyricum*.

MNO6 produced ethanol in very small amounts (0.018 mol/mol). Compared to the fermentation by the wild type, the ethanol yield was reduced by 68% and 89% from stored crude glycerol and technical grade glycerol, respectively. The biomass yield of MNO6 was also significantly reduced, constituting only 90% of the wild type. The reduced amount of biomass diminishes the need for ATP. By extrapolating the ATP yield from the mass balances (both in this study and from Jensen et al. [2012] (data not shown)) it is clear that also the ATP yield is reduced by 10% in MNO6. The reduced ATP requirement/production may be causing the shift in product pattern, as observed in this study.

In a fed-batch fermentation with MNO6 and *in situ* removal of butanol, 1,3-PDO would accumulate in the reactor, reaching high titers critical for downstream processing. Recently, the discovery of a bacterial strain producing both 1,3-PDO and ethanol from crude glycerol has been published (Rossi et al. [2012]). In order to reach high 1,3-PDO titers, the concentration of the second product, ethanol will also increase and possibly inhibit the organism. The fermentation by MNO6 only leads to small amounts of ethanol but high concentrations of butanol, which is even more toxic than ethanol. However, as butanol can be removed simultaneously by gas-stripping, it is possible to achieve high 1,3-PDO titers.

In this study, we have demonstrated that our mutant strain of *C. pasteurianum* can tolerate high concentrations of crude glycerol, has a high glycerol utilization rate, and high productivity of butanol and 1,3-PDO. Based on these results and on the results on non-treated crude glycerol, we consider MNO6 a more robust and more efficient strain than the wild type and, therefore, also better suited for industrial applications.

## Competing interests

The authors declare that they have no competing interests.

## Authors’ contributions

TØJ was main responsible for planning and performing the experiments, and drafted the manuscript. TK and MJM participated in planning and interpretation of experiments. PW participated in interpretation of experiments and finishing of the manuscript. All authors read and approved the final manuscript.
